# Antibodies for Assessing Circadian Clock Proteins in the Rodent Suprachiasmatic Nucleus

**DOI:** 10.1371/journal.pone.0035938

**Published:** 2012-04-27

**Authors:** Joseph LeSauter, Christopher M. Lambert, Margaret R. Robotham, Zina Model, Rae Silver, David R. Weaver

**Affiliations:** 1 Department of Psychology, Barnard College of Columbia University, New York, New York, United States of America; 2 Department of Neurobiology, University of Massachusetts Medical School, Worcester, Massachusetts, United States of America; 3 Department of Psychology, Columbia University, New York, New York, United States of America; 4 Department of Pathology and Cell Biology, Columbia University Health Sciences, New York, New York, United States of America; Vanderbilt University, United States of America

## Abstract

Research on the mechanisms underlying circadian rhythmicity and the response of brain and body clocks to environmental and physiological challenges requires assessing levels of circadian clock proteins. Too often, however, it is difficult to acquire antibodies that specifically and reliably label these proteins. Many of these antibodies also lack appropriate validation. The goal of this project was to generate and characterize antibodies against several circadian clock proteins. We examined mice and hamsters at peak and trough times of clock protein expression in the suprachiasmatic nucleus (SCN). In addition, we confirmed specificity by testing the antibodies on mice with targeted disruption of the relevant genes. Our results identify antibodies against PER1, PER2, BMAL1 and CLOCK that are useful for assessing circadian clock proteins in the SCN by immunocytochemistry.

## Introduction

The suprachiasmatic nucleus (SCN) of the mammalian hypothalamus generates daily rhythms in behavior, hormones and physiology. The SCN is composed of a heterogeneous population of neurons which form a functional circadian clock as a result of coupling through neurochemical interactions [1], [2]. Individual cells from SCN and from many other tissues express 24-hour molecular rhythmicity that results from a transcriptional-translational feedback loop. The transcription factors, CLOCK and BMAL1, form heterodimers and bind to E-box elements in the promoters of *Period* (*Per*) *1* and *Per2*, *Cryptochrome* (*Cry*) *1* and *Cry2* genes. The protein products of these genes form complexes, which translocate into the nucleus and interact with the CLOCK/BMAL1 complex, resulting in repression of their transactivational activity [3], [4]. Post-translational events modify the timing of this negative feedback, providing fine control over cycle length of the molecular oscillations [5]–[10].

Several circadian clock proteins, most notably PER1 and PER2, have high-amplitude rhythms of abundance in the SCN. Assessment of these molecular rhythms in the SCN is critical for understanding the effect of light, drugs, and other interventions on the phase of the SCN oscillator, and for delineating the impact of mutations affecting circadian clock function. Several previous studies have described circadian clock protein rhythms in the SCN [11]–[18]. However, it is often difficult to find antibodies that reliably label clock proteins, and for many commercially available antibodies, appropriate validation is not available [19].

The goal of this project was to generate and characterize antibodies against several circadian clock proteins. To this end, we generated and tested antibodies raised against PER1, PER2, CLOCK, and BMAL1 in the SCN of mice and hamsters, identifying antibodies that detect these antigens in the rodent SCN, and which promise to be useful for other studies of circadian clock gene products. We did not attempt to quantify the data or compare differences in staining among antibodies as the various antibodies were tested at different times. Instead, the results are a qualitative description of the staining quality obtained for each antibody studied using three criteria to determine sensitivity and selectivity with which each antibody appeared to label the intended antigen. First, immunoreactivity was expected to be more concentrated in the SCN than in surrounding areas based on gene expression profiles and other immunocytochemical studies. Second, SCN staining intensity was expected to be greater at ZT12 than at ZT0 for PER1 and PER2, as these antigens have previously been reported to have high-amplitude rhythmic expression in the SCN [11]–[18]. Third, immunoreactivity is expected to be absent in tissues from mice with targeted disruption of the corresponding gene.

## Methods

### Antigen sequences

The antigens were recombinant protein fragments, expressed in bacteria. cDNA constructs encoding fragments of mouse PER2, CLOCK, BMAL1 in the bacterial expression vector, Novagen pET-23b (EMD Biosciences, Gibbstown, NJ, USA), were generously supplied by Dr. Choogon Lee (Florida State University, Tallahassee, FL, USA), who previously used these constructs to generate antibodies to these proteins in rats and guinea pigs [5]. A cDNA fragment encoding a fragment of mouse PER1 was isolated by reverse-transcription PCR, and directionally subcloned into pET-23b. The sequence of the insert was verified in both directions. For each of these constructs, the pET-23b vector sequence encodes a 14-residue N-terminal epitope (MASMTGGQQMGRDP), followed by the circadian protein fragment fused in-frame with the vector sequence that includes the 6His epitope tag. More specifically, translation of the vector added the C-terminal amino acid sequence PNSSSVDKLAAALEHHHHHH to all proteins except CLOCK. For CLOCK, the C-terminal sequence was AAALEHHHHHH.

The antigen and residue numbers were as follows: for PER1 (PERIOD1, also called Rigui, BAA94086) residues 1–232 of 1291; for PER2 (PERIOD2, NP_035196) residues 1–200 of 1257; for BMAL1 (also known as ARNTL and MOP3; NP_031515), residues 382–580 (of 626); for CLOCK (AAC53200) residues 363–855 (of 855). In Lee et al. [5], these antigens were called PERIOD1-1, PERIOD2-1, BMAL1-2, and CLOCK-C.

### Antigen Expression and Purification

BL21(De3)pLysS bacteria were transformed, plated on selection ampicillin/chloramphenicol-containing selection plates and incubated overnight at 37°C. Single bacterial colonies were inoculated into 100 mL Autoinduction media containing ampicillin and chloramphenicol (Overnight Express Autoinduction System, Novagen). Cultures were grown overnight at 37°C with shaking. The cultures were then centrifuged (2,500 g, 15 minutes, 4°C), and the pellet was resuspended in guanidinium hydrochloride denaturing buffer, centrifuged (10,000 g, 20 minutes, 4°C), and the supernatant was incubated with Talon metal affinity resin (CLONTECH, Mountain View, CA, USA). After washing, samples were eluted from gravity columns with Denaturing Elution Buffer (CLONTECH) following the manufacturer's instructions. Samples were then dialyzed overnight in 8 M urea (Slide-A-Lyzer dialysis cassettes, Pierce Endogen). The purified proteins were visualized by staining samples separated on 12% SDS-PAGE gels with Coomassie blue (Biorad, Hercules, CA, USA).

### Regulatory Approvals for Animal Use

All animal studies were carried out in strict accordance with the recommendations in the Guide for the Care and Use of Laboratory Animals of the National Institutes of Health and Animal Welfare regulations. Immunization and bleeding of rabbits and guinea pigs for generation of antibodies was conducted at Cocalico Biologicals under protocols approved by their institutional animal care and use committee (protocols 040507CBISTD and 100407CBISTD). Care and use of Syrian hamsters and wild-type mice was conducted at Columbia University under protocols (AC-AAAB0480 and AC-AAAA9505) approved by the Columbia University Institutional Animal Care and Use Committee. Care and use of mutant and background-matched mice was conducted at the University of Massachusetts Medical School under protocols approved by the UMass Medical School Institutional Animal Care and Use Committee (protocol A-1572 and A-1315). For studies involving perfusion fixation, animals were deeply anesthetized with sodium pentobarbital. In all studies, every effort was made do minimize stress, discomfort, and pain of the experimental animals.

### Animals for Immunization

Antibodies were generated in rabbits and guinea pigs at Cocalico Biologicals, Inc. (Reamstown, PA, USA). Animals received an initial immunization in complete Freund's adjuvant, and subsequent booster immunizations in incomplete Freund's adjuvant. Boosts were administered at 1-month intervals, and blood samples were collected at 7–10 and 21 days after each boost. The number of boost-bleed cycles ranged from 2 to 8. For the final collections, animals were anesthetized and exsanguinated to collect a larger volume of blood. Blood samples were allowed to clot, centrifuged, serum samples were frozen, and shipped to UMMS. Samples were stored at −80°C. Dilutions refer to dilution of crude serum.

### Animals for Immunochemistry

#### Wild-type animals

Adult male C57BL/6 mice and LVG Syrian hamsters (Charles River Laboratories, Wilmington, MA, USA) were group housed (n = 3/cage) at Columbia University with room temperature at 21±1°C and *ad libitum* access to food and water. A dim red light (<1 lux) used for animal maintenance was on continuously. Mice were in a 12 hour light∶ 12 hour dark (12∶12 LD) cycle and were perfused at ZT 0 (the time of lights-on) or at ZT 12 (the time of lights-off). Hamsters were in a 14∶10 LD cycle and were perfused at ZT 0 (2 hrs before lights-on) or ZT12 (the time of lights-off).

#### Genetically modified mice and their genetic controls

Mice with targeted mutations of circadian clock genes were used to further validate the antibodies. Generation and characterization of the mutant lines has been previously reported [13], [17], [20]. Colonies were maintained at UMass Medical School. Mice were genotyped by PCR-based amplification of mouse genomic DNA and gel electrophoresis as previously described [13], [17], [20].

*Per1^−/−^* and *Per2^−/−^* mice were studied on an sv129 genetic background, while *Bmal1^−/−^* and *Clock^−/−^* mice were studied on a C57BL/6J genetic background. Mutant mice and control wild-type mice of the same genetic background were anesthetized and perfused within 2 hours of lights-out (ZT 12–14) and within 2 hours of lights-on (ZT 0–2) using the procedures described below. Brains were postfixed overnight, transferred to 20% sucrose, and then shipped to Columbia University for analysis.

#### *Per1^−/−^*

Mice with targeted disruption of *mPer1* were generated by Bae et al. [13]. The targeted allele removes exons 2 through 12 of the mouse *Per1* gene. This allele was initially called *mPer1^ldc^* and is more properly called *mPer1^tm1drw^*.

#### *Per2^−/−^*

Mice with targeted disruption of *mPer2* were also generated by Bae et al. [13]. The targeting event removes exon 5 and a portion of exon 6 of the mouse *Per2* gene. This allele was initially called *mPer2^ldc^* and is more properly called *mPer2^tm1drw^*. In some tissues of mice homozygous for this allele, a deletion mutant form of the PER2 protein is produced. Notably, this mutant protein was not detected in the SCN of these homozygous mutant mice by immunocytochemistry [13].

#### *Bmal1^−/−^*

Mice with targeted disruption of *Bmal1* (*Mop3* or *Arntl*) were generated by Bunger et al. [20]. Founder mice used to establish our colony were generously provided by Dr. C.A. Bradfield. Mice homozygous for the targeted allele are also called BMAL1-deficient mice.

#### *Clock^−/−^*

Mice with targeted disruption of the *Clock* gene (*Clock^Δ5–6^* allele, more properly called *Clock^tm1.1smr^*) were generated by DeBruyne et al. [17]. Mice homozygous for the targeted allele are also called *Clock^−/−^*, CLOCK-knockout (KO) or CLOCK-deficient mice.

In previous studies using other antibodies, the SCN of these mutant lines have been shown to lack immunostaining for the targeted gene in the SCN. More specifically, *Per1^−/−^* mice lack PER1 staining [13], *Per2^−/−^* mice lack PER2 staining [13], *Bmal1^−/−^* mice lack BMAL1 staining [15], and *Clock^−/−^* mice lack CLOCK staining in SCN [17].

### Perfusion and Fixation

Mice and hamsters were anesthetized (200 mg/kg pentobarbital, i.p.) at either ZT0 or ZT12. Following deep anesthesia, they were perfused intracardially with saline (50 or 100 ml for mice or hamsters, respectively) followed by 4% paraformaldehyde in 0.1 M sodium phosphate buffer (PB; 100 or 200 ml for mice or hamsters, respectively). Brains were removed and postfixed overnight, then cryoprotected in 20% sucrose for 2 days.

### Immunocytochemistry

Brain sections (50 µm) containing the SCN were cut on a cryostat at −20°C. Sections were washed 3 times for 10 min with 0.1 M PB containing 0.1% Triton-X-100, blocked for 1 hr with normal donkey serum (NDS) diluted 1∶50 in PB containing 0.3% Triton followed by incubation in the primary antibodies diluted in the same buffer. Pilot studies conducted with primary antibodies at 1∶5,000 dilution revealed that antibodies made in guinea pig generally gave more intense signal than antibodies made in rabbit. Therefore, primary rabbit antibodies were tested at concentrations of 1∶500, 1∶1,000 and 1∶5,000, while guinea pig antibodies were tested at 1∶1,000, 1∶5,000 and 1∶10,000. In cases where these concentrations gave strong background, the guinea pig antibodies were also tested at 1∶20,000 and 1∶40,000. After incubation of primary antibody for 48 hrs at 4°C, sections were washed twice for 10 min, once for 30 min, and once for 10 min in PB+0.1% Triton, and then were incubated for 2 hr in the appropriate secondary antibody (donkey anti-rabbit or donkey anti-guinea pig) conjugated to Cy2 fluorescent chromogen (Jackson ImmunoResearch, West Grove, PA, 1∶200 in PB+0.3% Triton). Sections were washed 3 times for 10 min in PB, mounted, dehydrated and coverslipped with Krystalon (EM Science, Gibbstown, NJ).

In some cases, one of two different amplification protocols was performed. In one, a biotinylated secondary antibody was used (donkey anti-rabbit or anti-guinea pig, 1∶200), followed by incubation in avidin-biotin peroxidase complex (ABC) for 1 hr (ABC Elite kit, Vector Laboratories, Burlingame, CA, USA; 40 µl/10 ml PB+0.3% Triton). In a second amplification protocol (ABC+BT), the biotinylated secondary antibody was followed by incubation in biotinylated tyramine (6 µl/10 ml 0.1 M PB+2 µl H_2_O_2_ for 30 min). Cy2 avidin (1∶200 in PB+0.3% Triton) was used as the fluorescent label.

### Analysis of Immunostained Sections

Sections were examined on a Nikon Eclipse E800 microscope (Nikon, Tokyo, Japan) using Plan-Apo 4×, 10× and 20× lenses with numerical aperture 0.2, 0.45 and 0.75 respectively (Nikon). Images were captured using QCapture software (version 2.95.0, Quantitative Imaging, Surrey, BC, Canada) connected to a Q-Imaging Retiga EXi, fast 1394 camera (Quantitative Imaging). To evaluate antibody quality and to control for inter-run variability, sections from mutant animals and their WT controls and those from ZT12 and ZT0 were processed simultaneously. Subsequently, images were captured under identical conditions. Images were imported into Photoshop 7.0 (Adobe Systems Inc., San Jose, CA, USA) and cropped to show the SCN. Shadows and highlights were adjusted in the Image-Adjustment-Levels dialog box, by dragging the black and white Input Levels to the edge of the first group of pixels on either end of the histogram.

## Results

**Figure 1:**
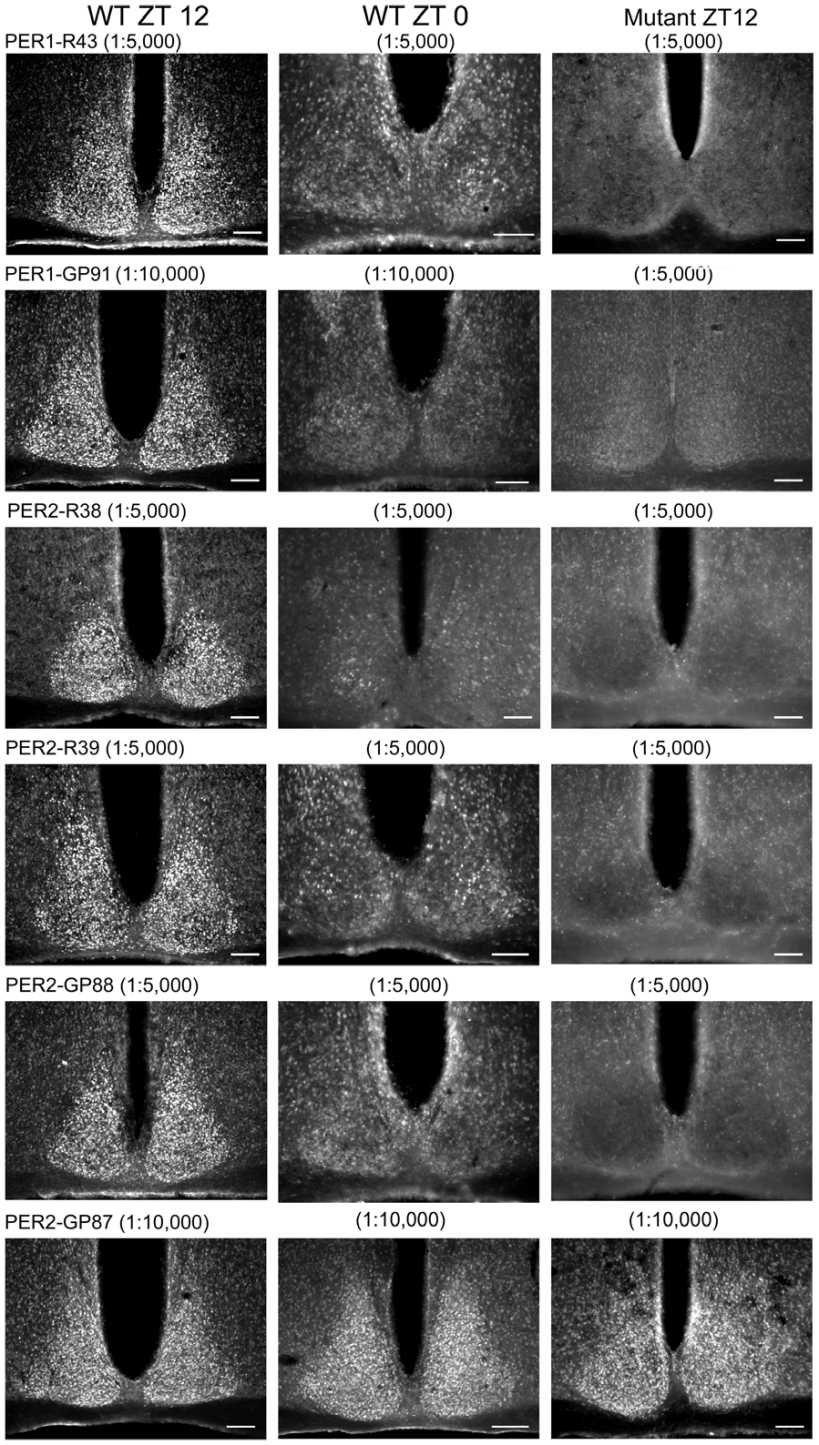
Photomicrographs of mouse SCN sections immunostained with PER1 and PER2 antibodies. The left column shows sections from wild-type mice euthanized at ZT12. The center column shows sections from wild-type mice euthanized at ZT0. The right column shows sections from mice lacking the target antigen (“mutant") at ZT12. The antigen, host number (R = rabbit; GP = guinea pig) and antibody concentration used for each antibody is indicated. Scale bar, 100 µm.

**Figure 2:**
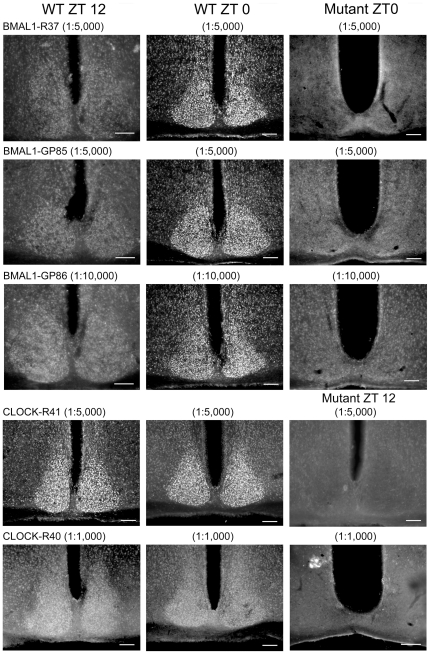
Photomicrographs of mouse SCN sections immunostained with BMAL1 and CLOCK antibodies. The left column shows sections from wild-type mice euthanized at ZT12. The center column shows sections from wild-type mice euthanized at ZT0. The right column shows sections from mice lacking the target antigen (“mutant") at ZT0 or 12, as indicated. The antigen, host number (R = rabbit; GP = guinea pig) and antibody concentration used for each antibody is indicated. Scale bar, 100 µm.

**Figure 3:**
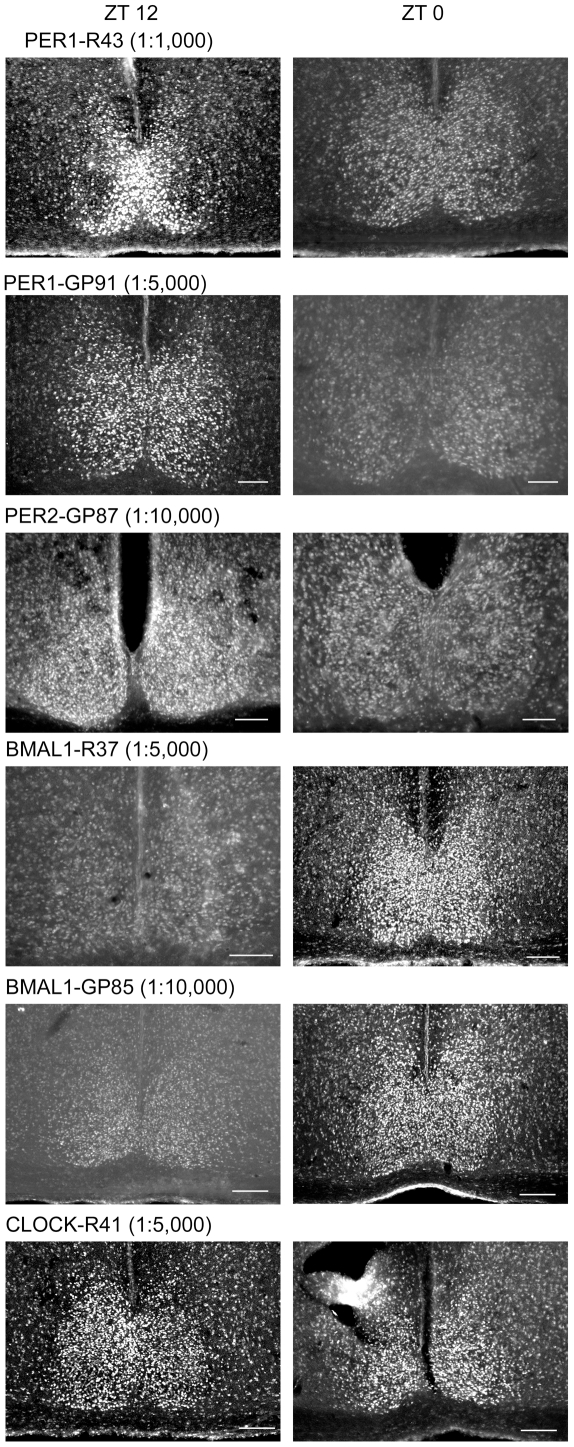
Photomicrographs of hamster SCN sections immunostained with PER1, PER2, BMAL1 and CLOCK antibodies. Columns show the staining at ZT12 (left) and ZT0 (right) level for each antigen. The antigen, host number (R = rabbit; GP = guinea pig) and antibody concentration used for each antibody is indicated. Scale bar, 100 µm.

Table 1 provides a list of all antibodies tested and the results of dilution series conducted at times when levels of the corresponding antigen were expected to be high in wild-type mice. Table 2 lists the results of studies conducted at the optimal dilution, comparing results at expected peak and nadir times, and the results of mutant mice examined at the peak time. Table 3 summarizes results from studies examining hamster SCN. Figures 1, 2, and 3 show photomicrographs of SCN staining for those antibodies producing strong SCN staining intensity at the concentrations that give the best labeling. Results for antibodies with poor staining are not shown in the figures.

**Table 1:** Assessment of Antibody Dilutions in Wild-Type Mice.

Antibody		Subjects		Antibody Dilutions			
Antigen	Host	Peak Time	N	1∶500	1∶1,000	1∶5,000	1∶10,000
PER1	R43	ZT12	4	+	+	+++	n.d.
PER1	R1177	ZT12	3	n.d.	n.d.	++	n.d
PER1	GP91	ZT12	3	n.d.	+	++	+++
PER2	R38	ZT12	4	+	++	+++	n.d.
PER2	R39	ZT12	5	+	++	+++	n.d.
PER2	GP87	ZT12	3	n.d.	+	++	+++
PER2	GP88	ZT12	5	n.d.	+	++	+
BMAL1	R36	ZT0	4	NS	NS	NS	n.d.
BMAL1	R37	ZT0	4	++	+++	+++	n.d.
BMAL1	GP85	ZT0	4	n.d.	+	+++	++
BMAL1	GP86	ZT0	4	n.d.	+	++	+++
CLOCK	R40	ZT12	3	+	+	+	n.d.
CLOCK	R41	ZT12	4	++	++	+++	n.d.

Host #: R = rabbit; GP = guinea pig. Peak Time: Wild-type mice were perfused at the time of expected peak of immunostaining and a series of dilutions was examined. N = number of animals tested. Results are scored as: −(no SCN cells labeled), +(relatively poor staining), ++(good staining in SCN), and +++(best staining of SCN cells compared to background). NS indicates non-specific staining (cells inside and outside the SCN were labeled with the same intensity). “n.d." indicates not determined.

**Table 2:** Summary of Immunostaining Results in Wild-type and Mutant Mice.

Antibody			Wild-type (Peak)		Wild-type (Nadir)		Mutant (Peak)	
Antigen	Host	Dilution	N	Staining	N	Staining	N	Staining
PER1	R43	1∶5,000	4	+++	3	+	2	−
PER1	GP91	1∶10,000	3	+++	2	+	2	−
PER2	R38	1∶5,000	4	+++	2	+	2	−
PER2	R39	1∶5,000	5	+++	2	+	2	−
PER2	GP87	1∶10,000	3	+++	2	+++	4	+++
PER2	GP88	1∶5,000	5	++	2	+	2	−
BMAL1	R36	1∶5,000	4	NS	2	NS	1	−
BMAL1	R37	1∶5,000	4	+++	2	+	1	−
BMAL1	GP85	1∶5,000	4	+++	2	+	1	−
BMAL1	GP86	1∶10,000	4	+++	2	+	1	−
CLOCK	R40	1∶5,000	3	+	2	+	2	−
CLOCK	R41	1∶5,000	4	+++	2	+++	2	−

Host #: R = rabbit; GP = guinea pig. Wild-type mice were perfused at the time of expected peak of immunostaining (ZT12 for PER1 and PER2; ZT0 for BMAL1), and at the expected low point (nadir) of immunostaining rhythms (ZT0 for PER1, PER2, and ZT12 for BMAL1). Mutant mice were examined at the time of peak staining in wild-type mice. Results from wild-type mice at peak time are reproduced from Table 1, for comparison. CLOCK immunostaining was comparable at ZT12 (arbitrarily called Peak Time) and ZT0 (arbitrarily called Nadir Time). CLOCK-deficient mice were examined at ZT12. N = number of animals tested. Results are scored as described in Table 1: −(no SCN cells labeled), +(relatively poor staining), ++(good staining in SCN), and +++(best staining of SCN cells compared to background). NS indicates non-specific staining (cells inside and outside the SCN were labeled with the same intensity).

**Table 3:** Summary of Immunostaining Results in Hamsters.

Antibody		Dilution Studies in Hamsters at Peak Time						Nadir Time	
Antigen	Host	Peak Time	N	1∶500	1∶1,000	1∶5,000	1∶10,000	N	Staining
PER1	R43	ZT12	3	++	++	+++	n.d.	3	+
PER1	R1177	ZT12	3	n.d.	n.d.	+++	n.d.	0	n.d.
PER1	GP91	ZT12	4	n.d.	+	+++	+	2	+
PER2	R38	ZT12	5	−	−	−	n.d.	0	n.d.
PER2	R39	ZT12	4	−	−	−	n.d.	0	n.d.
PER2	GP87	ZT12	3	n.d.	+	+	+++	2	+
PER2	GP88	ZT12	4	n.d.	−	−	−	0	n.d
BMAL1	R36	ZT0	3	−	−	−	n.d.	0	n.d.
BMAL1	R37	ZT0	2	n.d.	++	+++	++	2	+
BMAL1	GP85	ZT0	4	+	+	++	++	3	+
BMAL1	GP86	ZT0	3	NS	NS	NS	NS	3	NS
CLOCK	R40	ZT12	3	NS	NS	NS	n.d.	2	NS
CLOCK	R41	ZT12	3	+	++	+++	n.d.	3	+++

Host #: R = rabbit; GP = guinea pig. Peak Time: Hamsters were perfused at the time of expected peak of immunostaining and a series of dilutions was examined. Nadir Time: Hamsters were perfused at the expected low point of immunostaining rhythms (ZT0 for PER1, PER2, and ZT12 for BMAL1). CLOCK immunostaining was comparable at ZT0 and ZT12; dilution studies were conducted at ZT12 and results from ZT0 are listed arbitrarily as Nadir Time. Studies at nadir time used the antibody concentration found to give optimal labeling at the peak time. N = number of animals tested. Results are scored as Described in Table 1: −(no SCN cells labeled), +(relatively poor staining), ++(good staining in SCN), and +++(best staining of SCN cells compared to background). NS indicates non-specific staining (cells inside and outside the SCN were labeled with the same intensity). “n.d." indicates not determined.

For all effective antibodies, the following pattern of staining was observed. At high concentrations, each antibody produced relatively ubiquitous staining that was not enriched in the SCN. At lower concentrations, an optimal signal-to-noise ratio was achieved. At even lower concentrations, the intensity of staining was low everywhere.

### Antibodies Labeling Mouse SCN

#### PER1

Both R43 and GP91 worked well in mouse SCN (Fig. 1, Tables 1, 2).

R43 provided strong staining, specific to SCN cells at ZT12 when used at a 1∶5,000 dilution. At ZT 0, stained cells could be detected, but staining was much weaker than at ZT12 except for a few cells in the central SCN. No staining was detected with R43 in *Per1^−/−^* mice.

The GP91 antibody provided optimal staining in the SCN at ZT 12 when used at 1∶10,000. Staining was much paler at ZT0 except for a few cells in the central SCN. No staining was detected in *Per1^−/−^* mice. Higher concentrations of primary antibody produced more background staining for both R43 and GP91.

#### PER2

Three of the antibodies to PER2, namely R38, R39 and GP88 worked well. Used at 1∶5,000, each of these antisera produced strong signal in SCN cells at ZT 12, with pale staining at ZT0 in wild-type mice and no staining in *Per2^−/−^* mice at ZT 12. For R38 and R39 at ZT0, there were a few cells in the central SCN, while R39 and GP88 produced very pale cellular staining just outside the SCN. Higher concentrations produced more background staining for R38 and R39. For GP88, concentrations of 1∶1,000 gave very good signal but with higher background; at 1∶10,000, SCN cells were paler and barely distinguishable from peri-SCN areas. Labeling with antibody GP87 was not specific: GP87 gave strong immunostaining at both ZT12 and ZT0 and staining was present even in the SCN of *mPer2^−/−^* mice (Fig. 1, bottom row).

#### BMAL1

Three of the antibodies to BMAL1, R37, GP85 and GP 86 worked well (Fig. 2) while R36 gave no staining at any concentration used (Tables 1, 2). R37 and GP85 at 1∶5,000 and GP86 at 1∶10,000 strongly stained SCN cells at ZT 0 and produced very pale SCN staining at ZT12. No staining was detected in *Bmal1^−/−^* mice with any of these antisera. Additionally, all three antibodies produced pale cellular staining just outside the SCN at ZT0. For R37, concentration of 1∶500 gave high background; 1∶1,000 and 1∶5,000 gave comparable results (Table 1). GP 85 gave high background staining at 1∶1,000 and paler staining at 1∶10,000. GP86 gave high background staining at 1∶1,000 and 1∶5,000.

#### CLOCK

Antibody R41 worked well (Fig. 2). R41 used at 1∶5,000 strongly stained SCN cells at both ZT 12 and ZT 0. Cells in the peri-SCN were visible but paler than in SCN. R41 used at 1∶500 produced good staining of SCN cells, but background cells in the rest of the brain were also visible. The background brain cells were paler with 1∶1,000 and very pale with 1∶5,000. No staining was detected in *Clock^−/−^* mice.

Antibody R40 produced pale staining of SCN cells at 1∶1,000, and staining was paler still at 1∶5,000 at both ZT 12 and ZT 0. At 1∶500, background staining was high. No staining was detected in *Clock^−/−^* mice (Fig. 2, bottom row).

### Antibodies Labeling Hamster SCN

#### PER1

Both R43 and GP 91 antibodies worked well in hamster, with very strong SCN cellular labeling and pale peri-SCN cells staining at 1∶5,000 at ZT 12, and paler staining at ZT 0 (Fig. 3, Table 3). At concentrations of 1∶500 and 1∶1,000, R43 gave good staining of SCN cells, but with higher background. For GP91, 1∶500 gave good staining for SCN cells, but with high background while 1∶10,000 gave very pale SCN staining.

#### PER2

In hamster SCN, R38 and GP88 gave no positive staining at any concentration tested (Table 3). For R39, concentrations of 1∶500, 1∶1,000 and 1∶5,000 gave poor staining, and amplification with ABC or ABC+ biotinylated tyramine did not yield improvements. In contrast to the results in mice, the GP87 antibody seemed to work well in hamsters (see Discussion for further consideration of species differences). At 1∶10,000 GP87 gave strong immunostaining throughout the SCN at ZT12, and paler staining at ZT0 (Fig. 3). At concentrations of 1∶1,000 and 1∶5,000 it gave high background staining (Table 3).

#### BMAL1

Antibody R37 worked very well, and GP85 gave good staining in hamster SCN (Fig. 3). R37 stained SCN cells well at 1∶5,000. Pale peri-SCN cells were visible at both ZT0 and at ZT 12. A concentration of 1∶1,000 of R37 also gave good staining, but more extra-SCN cells were visible. A concentration of 1∶10,000 produced very pale staining of extra-SCN cells, but also reduced staining of SCN cells.

GP85 antibody stained SCN cells at 1∶10,000, although cells throughout brain were also labeled, but with somewhat less intensity than SCN cells at this concentration. Very pale staining was observed in SCN at ZT 12. The 1∶10,000 concentration of GP85 gave the best difference between SCN and non-SCN cells. Concentrations of 1∶20,000 and 1∶40,000 did not eliminate pale staining of brain cells outside the SCN, but gave reduced SCN staining.

GP86 stained SCN cells but also all other brain cells with the same intensity at all concentrations. R36 produced no positive staining (Table 3).

#### CLOCK

R41 worked well, while R40 was not specific in hamster SCN. R41 used at 1∶5,000 produced good staining of SCN cells at both ZT12 and ZT 0. Paler background extra-SCN cells were also visible. At lower concentrations, SCN cells are well labeled but were barely distinguishable from the extra-SCN brain cells. R40 stained all brain cells with similar intensity at both ZT12 and ZT0 (Table 3).

## Discussion

The lack of good antibodies makes it difficult to assess the impact of circadian time, phase shifting stimuli, or clock gene mutations on clock gene protein products. While there are commercially available antibodies, many have not been subjected to validation to assess their usefulness in immunocytochemistry. Here we generated several antibodies against the clock proteins PER1, PER2, BMAL1 and CLOCK, and tested them for staining in the SCN of both mice and hamsters (Tables 1, 2, and 3), identifying several that gave excellent results (Figs. 1, 2, and 3).

**Figure 4:**
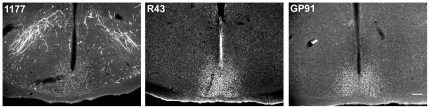
Photomicrographs of hamster SCN sections immunostained with PER1 antibodies. PER1 antibodies R1177, R43 and GP91 were used at a concentration of 1∶5,000. Animals were euthanized at ZT12. Scale bar, 100 µm.

A previously described PER1 antibody, generated in rabbit to the amino terminus of mouse PER1 (residues 6–21), called 1177, has been used successfully in many publications [11]–[15], [17], [18], [20]–[35]. This antibody gives good labeling of SCN cells in mouse, and no staining in *Per1^−/−^* mice [13]. In the hamster, 1177 stained SCN cells at about the same intensity as R43 and GP91 when used at the same concentrations. The 1177 antibody labels cells and fibers of the magnocellular neurons in the paraventricular and supraoptic nuclei, as previously described [30], while R43 and GP91 do not (Fig. 4).

For PER2, R38 and R39 antibodies produced good results in mouse SCN. Neither of these antibodies worked in hamster SCN. R38 has also worked in Nile grass rats (*Arvicanthis niloticus*) [27], [36] and lab rats (*Rattus norvegicus*) [37], [38].

The PER2 antibody GP87 produced non-specific staining in mouse, as it labeled SCN cells with the same intensity at ZT0 and 12, and staining intensity was not reduced in the SCN of *Per2^−/−^* mice (Figure 1, Table 2). While GP87 appeared to label hamster SCN cells strongly at ZT12 and weakly at ZT0, in view of the lack of specificity seen in mouse, this staining may not be specific to PER2 (see below for further consideration).

Although PER1 and PER2 expression are higher at ZT12 than at ZT0, some cells in the central SCN show high PER levels at ZT0 [11]–[13]. It may be that these cells express PER constitutively, or they may express PER in antiphase with the larger population [39], or the decline in PER expression in these cells may be delayed relative to the larger population [40].

CLOCK protein is generally thought to be constitutively expressed in the SCN [15], [41], although there is a report indicating that CLOCK is constitutively expressed in young but rhythmically expressed in old C57BL/6J mice [42]. Here, R41 worked in both mouse and hamster where it was highly expressed at both ZT0 and 12, suggesting constitutive expression. Immunostaining of the mouse SCN, and the absence of staining in *Clock^−/−^* mice, has recently been reported by another group using antibody R41 [43].

The previously published evidence for rhythmicity in BMAL1 protein levels is not consistent. Some studies describe rhythmic BMAL1 protein levels in the SCN, either with a nocturnal peak in rats (at ZT or CT14–22 in Wistar rats in LD or after 2 or 3 days in dim red light [44]), or with higher levels in daytime than nighttime in CD-1 mice in a 12∶12 LD cycle [41]. Other studies show no rhythm of BMAL1 staining in the SCN of C3H/HeJ mice kept for 1 day in dim red light after being in a 12∶12 LD cycle [15] or in young and old C57BL/6J mice [42]. Here, three antibodies to BMAL1 (R37, GP85, GP86) stained the mouse SCN well, while two of these antibodies (R37 and GP85) also stained the hamster SCN. In both species, with each of these antibodies, BMAL1 expression was high at ZT0 and low at ZT12, indicating a rhythm in BMAL1 in the SCN. Rhythmic expression of *Bmal1* RNA levels, with low levels during mid-day, has been reported in several studies; the phase of the BMAL1 protein rhythm reported here seems consistent with the 4–6 hr delay between transcript peak and protein peak typical of circadian clock proteins [3], [4]. Further analysis of BMAL1 protein rhythms is warranted.

While some antibodies labeled cells in extra-SCN regions, here we focused on SCN staining, as not all brain regions were tested for all antibodies and the time of peak expression in extra-SCN regions differs from the SCN [25]–[27], [45]. Nevertheless, antibodies producing robust signal in the SCN are likely to be useful for assessing protein rhythms in other neural sites.

Our systematic comparison of a set of polyclonal antisera to four key circadian proteins reveals some unexpected differences between the performance of these antisera in mouse and hamster. Most notably, three PER2 antisera useful in mouse SCN appear not to be useful in hamsters. As noted above, one of these PER2 antisera, R38, has been used successfully in the SCN and extra-SCN regions of Nile grass rats and lab rats [27], [36]–[38]. The single antibody that produced what appeared to be an SCN-specific signal with rhythmic labeling in the hamster was GP87. This antibody produced non-specific labeling in the mouse SCN, however, raising doubts as to its specificity in hamster as well. This species difference in PER2 staining with these antisera may be due to differences in the N-terminal 200 amino acid residues of the PER2 sequence used as the antigen. A 171-residue amino-terminal fragment of hamster PER2 [46] has 11 mismatches and requires 3 gaps to align with the mouse sequence it overlaps within the amino-terminal 200 residues. In contrast, the 200 amino-terminal residues of *Arvicanthis niloticus* PER2 align with the mouse sequence without gaps, and the sequences differ at only 8 residues (96% identity). Similarly, the lab rat PER2 sequence aligns to the mouse sequence without gaps and differs at only 7 residues (96.5% identity). Thus, despite the use of large (200 to 400 residue) fragments of the circadian proteins as immunogens (intended to produce a polyclonal response and maximizing the probability of reactivity in multiple species), the usefulness of these antisera in other species will need to be examined. Nevertheless, our study identifies a series of antibodies to these key circadian proteins that are useful in mice, and which may be useful in other species, possibly depending on the extent of species conservation of the amino acid sequences involved. An advantage of these antisera over some that are commercially available is that the antigens used here are clearly disclosed, so sequence alignment can be performed to predict potential reactivity in other species. (Four antibodies described here, PER1-R1177, PER2-R38, CLOCK-R41 and BMAL1-GP85 are commercially available through Millipore Corporation as product numbers AB2201 through AB2204). A further advantage is our use of genetically modified mice and two time-points with expected differences in protein expression levels, allowing functional assessment of the antibodies' performance. While each lab will need to verify the utility of these antibodies for their own species of choice and staining protocol, the results reported here indicate a starting point that should facilitate other work on immunocytochemical localization of circadian clock proteins in neural tissues.
